# Cardiopulmonary Exercise Testing in the Prognostic Assessment of Heart Failure: From a Standardized Approach to Tailored Therapeutic Strategies

**DOI:** 10.3390/medicina61101770

**Published:** 2025-09-30

**Authors:** Fiorella Puttini, Beatrice Pezzuto, Carlo Vignati

**Affiliations:** 1 Department of Critical Cardiology and Rehabilitation, Centro Cardiologico Monzino, IRCCS, 20138 Milan, Italybeatrice.pezzuto@cardiologicomonzino.it (B.P.); 2 Department of Cardiology, Tor Vergata Hospital of Rome, University of Rome “Tor Vergata”, 00133 Rome, Italy; 3 Department of Clinical Sciences and Community Health–Cardiovascular Section, University of Milan, 20122 Milan, Italy

**Keywords:** cardiopulmonary exercise testing, heart failure, peak VO_2_, VE/VCO_2_ slope, risk stratification, MECKI score

## Abstract

Cardiopulmonary Exercise Testing (CPET) is the gold standard for the functional assessment in patients with heart failure (HF), providing objective parameters that reflect the integrated response of the cardiovascular, respiratory, and muscular systems, in addition several CPET-derived variables have shown independent prognostic value in patients with both reduced (HFrEF) and preserved ejection fraction (HFpEF) HF. This review aims to critically analyze the main CPET prognostic variables in heart failure, highlighting their underlying pathophysiological mechanisms, their predictive capacity for mortality and hospitalizations, and their integration into clinical decision-making models. Parameters such as peak oxygen uptake (VO_2_), minute ventilation/carbon dioxide production (VE/VCO_2_) slope, periodic breathing (or exercise oscillatory ventilation—EOV), anaerobic threshold (AT), oxygen pulse, and VO_2_/work slope provide complementary insights into clinical risk; moreover, the combination of multiple CPET variables allows for more accurate risk stratification compared to the isolated use of each parameter. Multiparametric prognostic models such as the Metabolic Exercise Cardiac Kidney Index (MECKI) score, the Seattle Heart Failure Model, and the Heart Failure Survival Score (HFSS) incorporate these variables alongside clinical and laboratory data to guide advanced management and therapeutic decisions, including heart transplantation or left ventricular assistant device (LVAD) implantation. For these reasons, CPET-derived variables are essential prognostic tools in heart failure. Beyond improving risk stratification, their integration into multiparametric models supports a more personalized therapeutic approach, including tailored pharmacological management.

## 1. Introduction

Heart failure (HF) is a clinical syndrome characterized by structural and/or functional cardiac abnormalities associated with typical signs (e.g., peripheral edema, jugular vein distension) and/or symptoms (e.g., dyspnea) [1]. According to the latest European Society of Cardiology (ESC) guidelines, HF is classified into three categories: heart failure with reduced left ventricular ejection fraction (HFrEF; LVEF ≤ 40%), mildly reduced ejection fraction (HFmrEF; LVEF 41–49%), and preserved ejection fraction (HFpEF; LVEF ≥ 50%) [1].

Due to general population aging, improved diagnostics and better management of acute cardiovascular diseases, HF represents a growing clinical challenge with a substantial impact on mortality, morbidity, and healthcare costs. Despite therapeutic advances, particularly over the past decade, the prognosis of HF—especially in patients with HFrEF—remains poor [2].

Prognostic stratification plays a crucial role in the management of HF, as it allows clinicians to identify patients at higher risk of adverse events, personalize follow-up intensity, select candidates for advanced therapies (such as heart transplantation or mechanical circulatory support), and tailor pharmacological interventions accordingly [3].

**Figure 1:**
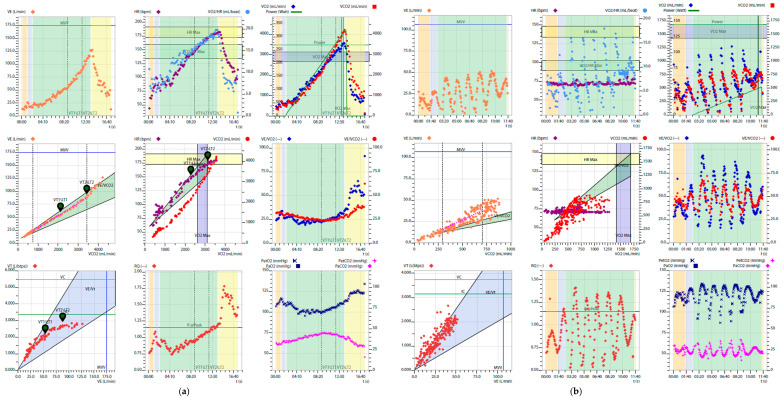
Examples of Wasserman nine plots. (**a**) Healthy subject; (**b**) Patient with severe HF, with low peakVO_2_, VO_2_/work slope, and O_2_ pulse, very high VE/VCO_2_ slope, and EOV.

In this context, cardiopulmonary exercise testing (CPET) is a valuable tool for functional assessment, though still underutilized except in specialized centers. Unlike other diagnostic tools, CPET offers a holistic view of heart, lungs, and muscles response to physical exertion. The test is based on the analysis of exhaled gases, from which key ventilatory and metabolic variables are derived. These are typically presented in standardized nine-panel plots, which integrate the main physiological responses, including oxygen uptake (VO_2_), carbon dioxide output (VCO_2_), minute ventilation (VE), ventilatory efficiency (VE/VCO_2_ slope), the VO_2_–work rate relationship, oxygen pulse (VO_2_/HR), tidal volume relative to ventilation, respiratory frequency, and end-tidal CO_2_ and O_2_ pressure (PetCO_2_ and PetO_2_) [4] (Figure 1). Beyond these variables, CPET also allows the identification of abnormal breathing patterns, which provide additional diagnostic and prognostic information in heart failure [5].

Notably, absolute contraindications to CPET are extremely rare [6], and the test can be performed safely even in highly specific and complex clinical scenarios, such as in tracheostomised patients [7].

In recent years, growing awareness of the complex and multifactorial causes of exercise intolerance in heart failure has driven the development of prognostic models that combine CPET-derived variables with clinical and laboratory findings. Among these, the Metabolic Exercise Cardiac Kidney Index (MECKI) score, the Heart Failure Survival Score (HFSS), and the Seattle Heart Failure Model (SHFM) have emerged as valuable tools for individualized risk assessment [3,8,9].

These models underscore the importance of a global and multidimensional approach, in which CPET-derived variables play a crucial role by adding significant prognostic value, as extensively demonstrated in the MECKI score, and by enhancing the accuracy of risk prediction compared to evaluating single parameters alone [10,11].

International guidelines on heart transplantation (ISHLT) emphasize that, in circumstances where candidacy for listing is uncertain, prognostic scores estimating a 1-year survival below 85% can provide valuable support in guiding the decision-making process [12]. In this context, CPET-derived variables—particularly peak VO_2_ and ventilatory efficiency—are integral to multiparametric models such as the MECKI score, which have proven effective in refining outcome prediction and supporting timely referral to advanced therapies.

Altogether, these aspects illustrate how CPET-derived parameters, when incorporated into multiparametric scores, can refine prognostic evaluation and support individualized treatment decisions.

The following sections provide a comprehensive appraisal of the main CPET variables and their integration into validated prognostic models, with a focus on their practical relevance in contemporary heart failure management.

## 2. Prognostic Variables Derived from CPET

### 2.1. Peak VO_2_

Peak VO_2_ is the maximum amount of oxygen an individual can consume during incremental exercise to the point of exhaustion [13]. It represents an integrated index of global functional capacity and is considered the gold standard for objectively estimating exercise tolerance. This parameter reflects the combined interaction between alveolar ventilation, oxygen diffusion, blood transport capacity, and peripheral oxygen utilization at the mitochondrial level [4]. According to the Fick principle, oxygen consumption (VO_2_) results from the product of cardiac output and the arteriovenous oxygen difference (ΔC(a–v)O_2_), thus encompassing both central and peripheral components of oxygen delivery and utilization [14].

The pathophysiological mechanisms underlying HF, such as reduced cardiac output, altered peripheral vascular response, and musculoskeletal dysfunction, are the main responsible factors for an early reduction in peak VO_2_, which can decrease by as much as 30–50% compared to age- and sex-adjusted normal values [15]. Its measurement overcomes the subjective limitations of the NYHA classification and serves as a robust prognostic indicator validated in multiple cohorts.

One of the earliest functional classifications based on peak VO_2_ was proposed by Weber and Janicki, defining four progressive stages (A–D): A (>20), B (16–20), C (10–16), and D (<10 mL/kg/min) [16].

Later on, Mancini et al. demonstrated that a peak VO_2_ value below 14 mL/kg/min is associated with increased 1-year mortality, supporting its inclusion among the eligibility criteria for heart transplantation [17]. More recent data from the MECKI Score Group have highlighted how beta-blocker therapy significantly modifies the prognostic interpretation of CPET variables. In particular, patients receiving high-dose beta-blockers (>25 mg/day of carvedilol-equivalent) exhibited improved survival outcomes regardless of their peak VO_2_ classification. As a result, transplant evaluation protocols have adopted revised VO_2_ thresholds—lowering the cut-off to <12 mL/kg/min in patients on beta-blocker therapy—to better reflect the survival benefit associated with optimized medical management [18].

Peak VO_2_ has also been validated as a surrogate endpoint in clinical trials. The HF-ACTION trial demonstrated that peak VO_2_, percentage of predicted VO_2_, and exercise duration are independent predictors of mortality and hospitalization in HFrEF patients, with a progressively increasing risk associated with lower values [19,20]. Through a sub-analysis of the HF-ACTION, Swank et al. demonstrated that peak VO_2_ has prognostic value not only for its absolute value but also for its variations over time: an increase of even just 1 mL/min/kg over three months is associated with significant clinical improvement in patients with HF, underscoring its usefulness as a dynamic marker of response to treatment [21]. Moreover, beside peak VO_2_, also VO_2_ at the anaerobic threshold (<11 mL/kg/min) have prognostic significance [22]. This represents a finding of notable clinical utility, particularly in patients who are unable to perform a maximal test (RER <1.1). While the association between peak VO_2_ and HFrEF has been widely validated, its prognostic value is increasingly being recognized in patients with HFpEF and HFmrEF as well. In this population percent-predicted VO_2_ (ppVO_2_) is increasingly used to assess prognosis [23]. Although no formal threshold has been universally adopted, values above 50% of predicted are generally associated with better outcomes, while lower percentages tend to reflect higher clinical risk [24].

This supports the notion that functional impairment, rather than systolic dysfunction alone, plays an essential role in disease progression. Ultimately, peak VO_2_ is responsive to therapeutic interventions, including pharmacological treatments, rehabilitation, and advanced procedures (e.g., cardiac resynchronization therapy—CRT, left ventricular assistant device—LVAD) [21]. However, its interpretation must consider confounding factors such as pulmonary comorbidities or obesity, which may distort its clinical meaning [25].

### 2.2. VE/VCO_2_ Slope

The VE/VCO_2_ slope (ventilatory efficiency slope) represents the relationship between minute ventilation (VE) and carbon dioxide production (VCO_2_) during incremental exercise. An elevated slope indicates increased ventilatory inefficiency, characterized by a disproportionate rise in ventilation relative to CO_2_ production, often accompanied by decreased PaCO_2_ [26].

In HF patients, the alteration of the VE/VCO_2_ slope is multifactorial. It primarily results from increased dead space ventilation, ventilation/perfusion (V/Q) mismatch, and heightened stimulation of pulmonary J-mechanoreceptors, triggered by abnormal vascular distension in the setting of pulmonary congestion [27]. A low cardiac index may also contribute to slope alteration due to reduced transpulmonary flow and impaired alveolar perfusion [28].

The VE/VCO_2_ slope is also associated with invasive hemodynamic parameters, such as mean pulmonary artery pressure, pulmonary capillary wedge pressure, and cardiac output [29,30,31], and it serves as a noninvasive surrogate marker of systemic congestion. Moreover, elevated slope values are inversely correlated with right ventricular ejection fraction and directly associated with pulmonary vascular resistance, particularly in patients with HFrEF [32,33].

From a clinical perspective, VE/VCO_2_ slope thresholds include: >34 considered abnormal and associated with unfavorable prognosis; 36–40: indicates high risk of adverse events; >45: strongly predictive of poor outcomes and used in selection criteria for heart transplantation or mechanical circulatory support (MCS) [34].

Multiple studies have demonstrated the superior prognostic value of the VE/VCO_2_ slope compared to peak VO_2_ for risk stratification in HF patients [28,35]. This parameter is less influenced by maximal effort and shows better reproducibility in the same patient [28].

The multicenter study by Corrà et al. which included 600 HFrEF patients followed for approximately 2 years, showed that VE/VCO_2_ slope was the most powerful independent predictor of major events (death or transplant), even among patients with intermediate peak VO_2_ (10–18 mL/kg/min). In this subgroup, a VE/VCO_2_ slope ≥ 35 identified a high-risk group (mortality ~30%), comparable to those with peak VO_2_ ≤ 10 mL/kg/min [35].

In a subanalysis of the REVIVAL registry, VE/VCO_2_ slope emerged as the strongest submaximal predictor for identifying patients at risk of death, transplant, or LVAD implantation within one year. In particular this parameter improves significantly after LVAD implantation, which is probably due to increased cardiac output and improved pulmonary perfusion [36,37].

Recent insights have also highlighted the clinical utility of the VE/VCO_2_ Y-intercept, a parameter that reflects dead space ventilation (VD). Gargiulo et al. demonstrated that increasing anatomical dead space during exercise proportionally increases the VE-axis intercept, confirming its close relationship with VD and suggesting its potential as a non-invasive estimator [38]. Apostolo et al. further validated its diagnostic value, showing that VEint values ≥ 4.07 L/min reliably identify HF patients with coexistent chronic obstructive pulmonary disease (COPD) [39]. Importantly, while the VE/VCO_2_ slope may appear normal—or even decreased—in the presence of mechanical ventilatory constraints such as those seen in COPD, the intercept increases significantly due to higher dead space. This apparent dissociation underscores the complementary diagnostic value of assessing both the slope and the intercept in HF populations, particularly in cases with overlapping pulmonary pathology.

Ventilatory inefficiency has also been recognized as a negative prognostic marker in HFpEF [40,41]. Although a direct causal relationship has not been established, it is likely related to diastolic dysfunction, where abnormalities in LV relaxation and compliance compromise ventricular filling and reduce exercise capacity, even with preserved systolic function [42,43].

A recent editorial by De Martino and Agostoni discussed how alveolar dead space increases in HFpEF patients under effort, resulting in progressive ventilatory inefficiency. This phenomenon is detectable through a CPET-derived combination of an elevated VE/VCO_2_ slope and a Y-intercept close to zero or even negative. While the slope reflects the amount of ventilation needed per unit of CO_2_ exhaled, the Y-intercept serves as an indirect indicator of dynamic changes in dead space ventilation during exercise [44].

Thus, CPET provides a valuable non-invasive window into the cardio-pulmonary limitations present in HFpEF, complementing echocardiographic and hemodynamic assessments and potentially guiding tailored therapeutic strategies.

### 2.3. Ventilation

During exercise, ventilation (VE) is finely regulated to ensure adequate gas exchange and maintain stable arterial carbon dioxide pressure (PaCO_2_). At low and moderate workloads, the ventilatory response is mainly determined by PaCO_2_ control, whereas at higher intensities, the increase in tidal volume (VT) relative to dead space (VD) and the development of lactic acidosis contribute to a further rise in CO_2_ release [26,27]. In heart failure, these mechanisms are often impaired: increased wasted ventilation [45,46], abnormal activation of chemo- and metaboreceptors [47], and the early occurrence of acidosis [48] may all contribute to ventilatory inefficiency. In addition, patients with heart failure often exhibit a restrictive pulmonary pattern, due to interstitial congestion and cardiomegaly related lung compression [49,50,51]. These structural factors limit tidal volume expansion, further increasing the ventilatory cost of exercise and worsening dyspnea and effort intolerance.

Beyond the disease itself, pharmacological therapy can also modulate the ventilatory response. In this context, the CARNEBI trial (CARvedilol vs. NEbivolol vs. BIsoprolol) performed a multiparametric comparison of the three most widely used β-blockers in heart failure [52]. This cross-over study in patients with moderate HF demonstrated that:

- Carvedilol was associated with a reduction in alveolar-capillary diffusion capacity (DLCO), likely due to an adverse effect on the membrane component, but at the same time provided better ventilatory efficiency during exercise, probably through modulation of chemoreflex control.
- Nebivolol and Bisoprolol, both β1-selective, better preserved pulmonary diffusion capacity and peak exercise performance, although they showed a less favorable ventilatory profile compared with Carvedilol.

Overall, the trial highlighted that β-blockers with different pharmacological profiles can selectively influence both cardiopulmonary function and ventilatory response. These findings emphasize the importance of a personalized therapeutic choice, tailored not only to ventricular function but also to the patient’s respiratory and ventilatory profile [53].

### 2.4. Anaerobic Threshold

The anaerobic threshold (AT) represents the point during exercise at which aerobic metabolism becomes insufficient to meet energy demands, leading to a progressive activation of anaerobic metabolism. This metabolic shift is associated with increased lactate and carbon dioxide production, which in turn triggers a compensatory rise in minute ventilation [54]. In healthy individuals, AT occurs at 50–60% of VO_2_ max, reflecting good metabolic efficiency [55]. In patients with heart failure, AT is often reached at a lower VO_2_, reflecting reduced exercise tolerance. In this population, such intolerance commonly stems from a combination of reduced peripheral oxygen extraction, skeletal-muscle mitochondrial dysfunction, and inadequate cardiac reserve [56].

The oxygen consumption at AT (VO_2_@AT or VO_2_@VAT) is a significant predictor of cardiovascular mortality and hospitalization, especially in patients unable to reach maximal effort (RER < 1.1), where peak VO_2_ may be unreliable.

The study by Gitt et al. (2002) demonstrated that a VO_2_@VAT below 11 mL/kg/min, when combined with a VE/VCO_2_ slope above 34, identified patients with a fivefold increased risk of medium-term mortality compared to those above these thresholds. Notably, this combined risk was even more pronounced during the early follow-up period, with a nearly tenfold increase in mortality observed within the first 6 months [22].

In 2013, Agostoni et al. showed in a large cohort of over 3000 HFrEF patients that the absence of an identifiable VAT on CPET was associated with a 41% increased risk of cardiovascular mortality or transplantation, independently of height, weight, peak VO_2_, and traditional MECKI score variables [57].

While the identification of both AT and the respiratory compensation point (RCP)—commonly referred to as the “double threshold”—has long been recognized as a marker of physiological integrity during exercise, a multicenter study by Carriere et al. was among the first to evaluate its prognostic implications in a large HFrEF population. The RCP represents the point at which ventilation begins to increase disproportionately to carbon dioxide production, due to progressive metabolic acidosis and the need to buffer accumulating hydrogen ions. Their findings demonstrated that patients in whom both thresholds were identifiable had significantly better outcomes, while the absence of one or both thresholds predicted a higher risk of mortality, urgent heart transplantation, or LVAD implantation [58]. Moreover, the inclusion of this binary information enhanced the prognostic performance of traditional CPET parameters such as peak VO_2_ and VE/VCO_2_ slope, suggesting its utility as a simple and practical adjunct in clinical risk stratification, particularly in cases where absolute values may be inconclusive.

### 2.5. Exercise Oscillatory Ventilation (EOV)

Exercise oscillatory ventilation (EOV) or periodic breathing is a respiratory pattern characterized by regular cycles of hyperventilation followed by hypoventilation during cardiopulmonary exercise testing (CPET) [59]. It is distinct from Cheyne-Stokes respiration, which typically occurs at rest, although both patterns may coexist in patients with advanced heart failure [60].

EOV results from an instability in ventilatory control, primarily due to delayed signaling from central and peripheral chemoreceptors. This instability creates an oscillatory feedback loop between CO_2_ production and removal, leading to alternating phases of over-and under-ventilation [61]. This phenomenon is exacerbated in heart failure patients due to:

- Increased sensitivity to changes in arterial CO_2_ pressure (PaCO_2_),
- Reduced hemodynamic reserve,
- Elevated pulmonary capillary pressure,
- Stimulation of J receptors from pulmonary vascular congestion.

These factors contribute to the emergence of an oscillatory ventilatory pattern that reflects severe cardiopulmonary dysfunction.

Currently, no universally accepted definition of EOV exists, and the detection of EOV is performed via visual inspection of ventilatory tracings obtained during CPET. However, the most commonly adopted criteria define EOV as a persistent oscillatory pattern occurring for ≥60% of exercise duration, with an amplitude ≥15% relative to the resting mean value [62].

Early evidence of the prognostic relevance of EOV came from Corrà et al., who showed that EOV frequently coexists with severe central sleep apnea (CSA, defined as AHI ≥ 30/h), and that their combination identifies a subgroup of chronic heart failure patients at markedly higher risk of mortality than either condition alone [59]. These findings suggest that EOV may serve as a marker of broader respiratory instability in heart failure, beyond exercise-induced changes alone.

Extensive evidence has established the independent prognostic value of exercise oscillatory ventilation (EOV) in patients with heart failure with reduced ejection fraction (HFrEF) [63,64,65,66], and more recent data suggest its relevance also in those with mildly reduced ejection fraction (HFmrEF) [67,68].

Further confirmation came from Guazzi et al. (2019), who analyzed over 5700 patients from the MECKI score registry. EOV was observed in 17% of HFrEF and 16% of HFmrEF patients and was associated with significantly worse outcomes in both groups. In HFrEF, survival curves diverged early in the follow-up, while in HFmrEF the negative prognostic impact of EOV emerged more gradually, becoming evident after 18 months [67]. These data emphasize the utility of EOV even in phenotypes with less severe functional impairment.

Finally, Rovai et al. (2019), also within the MECKI project, confirmed that the inclusion of EOV in predictive models significantly improves their discriminative power compared to standard clinical and CPET variables alone [63]. EOV thus emerges as a strong physiological marker of advanced ventilatory and cardiac dysfunction, capable of anticipating clinical deterioration even in patients with apparently preserved exercise tolerance.

### 2.6. VO_2_/Work Rate (ΔVO_2_/ΔWR)

The relationship between oxygen consumption (VO_2_) and the increment in external workload (Work Rate, WR) during exercise reflects the integrated efficiency of the cardiovascular system, peripheral musculature, and aerobic metabolism. Under physiological conditions, VO_2_ increases linearly with each additional Watt of workload, with a typical slope around 10 mL/min/Watt [4]. A change in the slope during exercise in highly suggestive for reduced oxygen delivery [69,70].

In heart failure patients, a reduced VO_2_/WR slope (<8.0 mL/min/Watt) indicates an inadequate cardiovascular response to effort [15]. This parameter has demonstrated independent prognostic value and may be particularly useful in patients who prematurely terminate the exercise test, making peak VO_2_ unreliable [71].

### 2.7. VO_2_/Heart Rate (O_2_ Pulse)

The oxygen pulse (O_2_ Pulse) is defined as the ratio between oxygen consumption (VO_2_) and instantaneous heart rate (HR) during exercise. It serves as a surrogate marker of stroke volume, based on the Fick equation: VO_2_ = Cardiac Output (CO) × arteriovenous oxygen difference (Δ[a–v]O_2_), thus VO_2_/HR ≈ Stroke Volume × Δ(a–v)O_2_ [14,72].

In normal conditions, O_2_ Pulse rises steadily with increasing exercise intensity [4,15]. Early plateauing in the VO_2_/HR curve suggests an inability of the heart to augment stroke volume, typically due to left ventricular dysfunction, myocardial ischemia, or limited contractile reserve [73,74].

Thus, it is a reliable, non-invasive surrogate for assessing cardiac pumping capacity during exercise.

Multiple studies have shown that an abnormal O_2_ Pulse pattern is associated with higher mortality rates, increased risk of adverse events, and greater likelihood of heart transplantation or LVAD implantation [75,76,77].

Vignati et al. recently confirmed the strong correlation between O_2_ pulse and directly measured cardiac output during inert gas rebreathing maneuvers, reinforcing its role as a functional marker of central hemodynamics [78]. Furthermore, Mapelli et al. demonstrated that even in patients with hypertrophic cardiomyopathy, characterized by preserved LVEF, an abnormal O_2_ pulse kinetics may reveal latent contractile dysfunction and predict adverse outcomes [79]. These findings highlight the broader applicability of VO_2_/HR analysis in revealing subclinical pump failure, even beyond conventional heart failure phenotypes.

## 3. From Standard to Complex CPET: Methodological Insights

**Figure 2:**
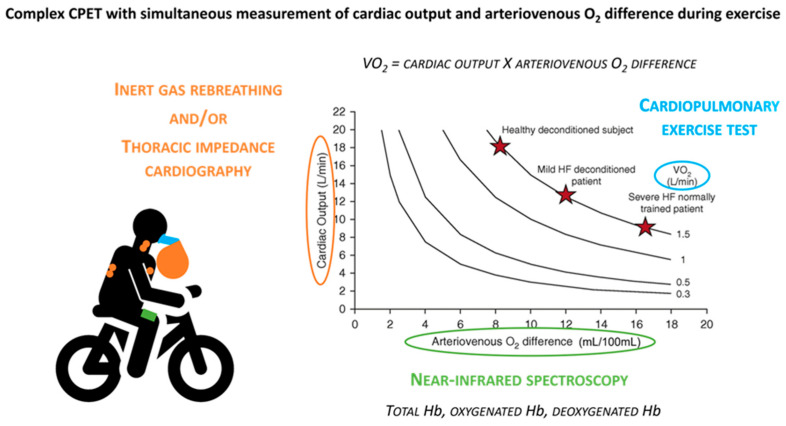
Complex CPET with simultaneous measurement of cardiac output and arteriovenous O_2_ difference during exercise. Reproduced with permission from ref. [13].

However, standard CPET does not allow the distinction between a reduction in VO_2_ due to impaired cardiac output (CO) or reduced peripheral oxygen extraction [ΔC(a–v)O_2_] [80]. To overcome this limitation, methods have been developed that integrate non-invasive CO measurement during the test. The main ones are inert gas rebreathing [81], which estimates CO by assessing pulmonary flow through the breathing of inert gases, and thoracic bioimpedance [82], which measures changes in the electrical impedance of the chest generated by the passage of blood; while near-infrared spectroscopy (NIRS) allows non-invasive assessment of muscle oxygenation and, therefore, the state of peripheral perfusion [83]. This integrated approach is commonly referred to as “complex CPET” as it combines standard cardiopulmonary exercise testing with additional hemodynamic and peripheral measurements to provide a more comprehensive evaluation of exercise limitation [13,84] (Figure 2).

In recent years, there has also been a growing awareness of the limitations of Fick’s law in clinical practice. Although the physiological principle remains valid, its application has often been based on estimated rather than measured VO_2_ values, with the risk of inaccurate CO_2_ assessment. In this sense, the study by Karsten and Vignati has made an important contribution by demonstrating that the calculation of CO based on estimated VO_2_ can deviate significantly from both the values obtained with direct measurement of VO_2_ in the haemodynamics laboratory [85]. This confirms that only direct measurement of VO_2_ during catheterization guarantees a clinically accurate result.

## 4. The Role of Multiparametric Scores Based on CPET

Although left ventricular ejection fraction (LVEF) remains a cornerstone in the classification of heart failure, its correlation with peak oxygen consumption (VO_2_) is limited, reflecting the fact that LVEF does not fully capture the complex pathophysiology underlying exercise intolerance [17,86,87].

This weak association highlights a fundamental limitation: LVEF primarily reflects systolic performance but fails to capture the integrated cardiopulmonary and peripheral adaptations that determine exercise capacity. In contrast, VO_2_ is a more comprehensive index that incorporates cardiac output, peripheral perfusion, oxygen extraction, and muscular efficiency.

Moreover, LVEF is an operator-dependent measure, influenced by image quality, interpretation variability, and loading conditions—factors that may reduce its reproducibility and reliability across centers [88]. Despite these limitations, LVEF continues to dominate therapeutic decision-making, often at the expense of more physiologically relevant parameters.

With the advent of modern pharmacological therapies—such as beta-blockers, angiotensin receptor–neprilysin inhibitors (ARNIs), sodium-glucose co-transporter 2 (SGLT2) inhibitors, and mineralocorticoid receptor antagonists (MRAs)—which improve outcomes independently of LVEF, reliance on this single static metric is increasingly inadequate [89,90]. The weak correlation between LVEF and VO_2_ reinforces the need for a more nuanced, multiparametric, and functionally oriented approach to risk stratification in HF patients, particularly when evaluating candidates for advanced interventions. For this reason, in recent years several prognostic scores have been developed and validated, integrating CPET-derived variables with clinical, laboratory, and imaging data to provide a more accurate and individualized risk assessment in both HFrEF and HFpEF populations. These tools aim to overcome the limitations of isolated metrics and to support therapeutic decision-making with greater precision.

### 4.1. MECKI Score

The MECKI score (Metabolic Exercise test data combined with Cardiac and Kidney Indexes) is currently the most comprehensive and validated prognostic score that integrates CPET-derived variables [10]. Developed from a large multicenter Italian registry, it combines six parameters: peak VO_2_, VE/VCO_2_ slope, left ventricular ejection fraction (LVEF), hemoglobin, serum sodium, and glomerular filtration rate (eGFR). It is available online and provides estimates of transplant- or LVAD-free survival at 2 years.

- According to published data, the MECKI score stratifies patients into four categories:
- <5% (906 patients)
- 5–10% (449 patients)
- 10–15% (236 patients)
- >15% (418 patients)

Kaplan–Meier survival curves showed a clear separation between groups, confirming the strong discriminative ability of the score. Moreover, ROC analysis demonstrated a high AUC (≈0.80 at 1–2 years), further supporting the predictive accuracy of the model [10].

The MECKI score has undergone extensive validation across different clinical contexts. Its prognostic accuracy was first confirmed by Corrà et al. in a large prospective Italian multicentre cohort, showing robust performance even in patients at relatively lower clinical risk [91].

Furthermore, in a large comparative study, Agostoni et al. (2018) demonstrated that the MECKI score outperformed other widely used prognostic models—including the Heart Failure Survival Score (HFSS) and the Seattle Heart Failure Model (SHFM)—showing superior discriminatory power at both 2- and 4-year follow-ups [3].

More recently, Adamopoulos et al. (2023) externally validated the MECKI score in an international multicentre cohort, including over 1000 patients from seven European countries and one Asian center, with predictive accuracy comparable to the original derivation population [92]. In this study, 844 patients with HFrEF were analysed: event-free survival was ~12 years for scores <10%, 9.5 years for 10–20%, and 2.8 years for ≥20%. The model showed excellent discrimination, with AUC values of 0.85 at 1–2 years and remaining >0.77 up to 10 years, further supporting its clinical implementation [92].

Recent findings from the MECKI score registry (Agostoni et al., 2023) have provided new insights into the prognostic evaluation of patients with heart failure and improved ejection fraction (HFimpEF)—a subset defined by recovery of LVEF from ≤40% to >40% over time. While this improvement in systolic function is often interpreted as a marker of favorable clinical evolution, the study demonstrated that LVEF recovery does not necessarily correspond to a full functional recovery or to a low-risk profile.

In this large multicenter cohort, many HFimpEF patients continued to exhibit reduced exercise capacity and abnormal ventilatory efficiency despite normalized or improved LVEF. In particular, peak VO_2_ and VE/VCO_2_ slope remained strong and independent predictors of adverse outcomes, including mortality and hospitalization [93]. These CPET-derived parameters consistently outperformed LVEF in risk stratification, highlighting the disconnect between resting systolic function and dynamic cardiorespiratory performance.

This emphasizes the need for a multiparametric evaluation: relying solely on imaging-based LVEF values may lead to underestimation of residual risk and misclassification of clinical status. The MECKI score proved effective in this population as well, underscoring its validity across different HF phenotypes, including those with apparent reverse remodeling.

Moreover, emerging data suggest that the prognostic interpretation of CPET parameters is not static over time. Paolillo et al. (2019) showed that the thresholds of peak VO_2_ and VE/VCO_2_ slope associated with specific risk levels have shifted over the past two decades, in parallel with improved overall prognosis due to the introduction of modern therapies (e.g., beta-blockers, ACE inhibitors, CRT, ARNI, SGLT2i) [94].

Additionally, Pezzuto et al. (2023) demonstrated that serial reassessment of the MECKI score provides incremental prognostic value beyond a single baseline evaluation. In a cohort of patients undergoing two CPET-based assessments at least six months apart, changes in MECKI score—particularly those driven by improvements or deteriorations in peak VO_2_ and VE/VCO_2_ slope—were strongly associated with subsequent outcomes. Patients whose MECKI score improved had significantly better prognosis than those whose score worsened, even when LVEF remained unchanged [95]. These results reinforce the utility of CPET-derived risk models not only for initial stratification but also for longitudinal monitoring and therapeutic guidance.

### 4.2. HFSS (Heart Failure Survival Score)

The Heart Failure Survival Score was among the first models to incorporate a CPET parameter -peak VO_2_. It combines this with six other variables: NYHA class, sinus rhythm, systolic blood pressure, FEV1, serum sodium, and LVEF.

Patients are categorized into three prognostic groups:

- Low risk: HFSS > 8.1.
- Intermediate risk: HFSS 7.2–8.09.
- High risk: HFSS ≤ 7.19.

In the original cohort, high-risk patients had a 1-year mortality rate of 50%, compared to 93% survival in low-risk individuals. However, the model does not include VE/VCO_2_ slope and tends to overestimate risk in patients treated with beta-blockers, limiting its current applicability in some settings [8].

### 4.3. SHFM (Seattle Heart Failure Model)

The Seattle Heart Failure Model (SHFM) is a widely used prognostic tool developed from a cohort of outpatients with chronic HF [9]. Although it does not include CPET variables directly, it integrates numerous clinical, laboratory, and pharmacological factors (age, sex, LVEF, creatinine, sodium, hemoglobin, use of ACE inhibitors, beta-blockers, statins, implantable cardioverter-defibrillators (ICDs), CRT, etc.).

It is often used alongside CPET to provide a multidimensional risk profile. The model estimates 1-, 2-, and 3-year survival and has shown excellent predictive calibration. In external validation, patients with higher SHFM scores had an annual mortality >20%, compared to <5% in those at lowest risk [96]. Although not CPET-based, its utility is well-recognized in pre-LVAD evaluation and therapeutic monitoring over time [97].

### 4.4. ISHLT Listing Criteria

The recommendations from the International Society for Heart and Lung Transplantation (ISHLT) do not constitute a numerical score but represent a decision-making framework based on clinical and physiological thresholds [12]. Among the CPET variables recommended for heart transplant listing are:

- Peak VO_2_ ≤ 14 mL/kg/min.
- Peak VO_2_ ≤ 12 mL/kg/min in patients on beta-blocker therapy.
- In patients with obesity (BMI ≥ 30 kg/m^2^): peak VO_2_ adjusted for lean body mass ≤ 19 mL/kg/min.
- For all patients—particularly when CPET is submaximal: VE/VCO_2_ slope > 35.
- In women or in patients aged ≤50 or ≥70 years: peak VO_2_ ≤ 50% of predicted.

In addition to these parameters, the ISHLT also emphasizes the potential value of prognostic score in cases where traditional criteria do not provide sufficient clarity. These scores, especially when the estimated 1-year survival is <85%, may support clinical decision-making, although they should not be used as standalone criteria for listing [12].

## 5. Discussion

This review confirms the strong prognostic value of CPET-derived variables in heart failure, particularly when evaluated in combination rather than in isolation. Parameters such as peak VO_2_, VE/VCO_2_ slope, anaerobic threshold, and O_2_ pulse each provide independent prognostic information, but their integration within multiparametric models significantly enhances sensitivity and accuracy in predicting clinical outcomes.

The comparison between the main available scores highlights significant differences. The HFSS, one of the earliest models developed, represented a pioneering step by including CPET-derived variables such as peak VO_2_, and for a long time served as a reference tool for selecting candidates for heart transplantation. However, its predictive capacity has proven limited in contemporary populations, where advanced pharmacological therapies are widely implemented [98].

In contrast, the SHFM was conceived as a clinical score based on demographic, clinical, and therapeutic variables, and has been extensively validated across different settings [99,100]. Attempts to improve its prognostic accuracy by adding CPET parameters, such as peak VO_2_, have shown only limited benefits, even though these variables have independent prognostic value [101,102].

More recent studies, however, have shown that the addition of specific dynamic parameters, such as EOV, can effectively enhance the prognostic performance of both HFSS and SHFM. This example underscores how the integration of CPET-derived variables, particularly those that capture dynamic functional responses, may refine and update traditional risk models, making them more suitable for contemporary clinical practice [103].

By contrast, the MECKI score has demonstrated more consistent prognostic superiority, thanks to the inclusion of key CPET variables (peak VO_2_ and VE/VCO_2_ slope) together with laboratory and clinical data. In large multicenter cohorts, the MECKI score showed high accuracy in predicting cardiovascular mortality, urgent transplantation, and LVAD implantation at both short- and long-term follow-up, confirming its reliability as one of the most valuable tools for guiding complex clinical decisions.

A particularly relevant aspect is that improvement in LVEF does not necessarily translate into a corresponding improvement in functional status. As demonstrated by Agostoni and colleagues in patients with HFimpEF, increases in LVEF may not be reflected in improved exercise capacity or overall prognosis. This finding further underscores the value of CPET as a tool capable of capturing true functional changes and integrating information that cannot be inferred from resting left ventricular function alone.

Looking ahead, techniques such as near-infrared spectroscopy (NIRS), inert gas rebreathing, and thoracic bioimpedance are increasingly used to complement CPET, broadening the framework of complex CPET. Although these methods are not yet part of standardized protocols or incorporated into established prognostic scores, they have already shown strong potential to deepen our understanding of the pathophysiological mechanisms of heart failure at the individual level [104,105,106]. By capturing aspects such as peripheral oxygen extraction, cardiac output, and ventilatory efficiency with greater precision, they may contribute to more accurate prognostic evaluation and provide valuable guidance for tailoring therapy to each patient.

Furthermore, emerging applications of machine learning, such as topological data analysis, have shown promise in combining CPET-derived variables with clinical, laboratory, and echocardiographic data to identify distinct patient clusters and divergent trajectories of disease progression [107]. The work of Agostoni and colleagues demonstrated how such multiparametric, AI-driven strategies can capture the heterogeneity of heart failure, identifying bifurcation points and terminal pathways associated with markedly different outcomes.

Together, these findings underscore the need to move beyond static risk markers and embrace dynamic, multidimensional tools, with CPET serving as a cornerstone of this evolving paradigm.

## 6. Conclusions

CPET-derived variables are not only reliable predictors of outcome in heart failure but also indispensable tools to personalize management, from tailoring pharmacological therapy to identifying the optimal timing for advanced interventions. By critically revisiting existing prognostic models, this review emphasizes that CPET should be considered a cornerstone of multiparametric risk assessment rather than an ancillary test. Future perspectives, including the integration of complex CPET and machine learning, will further enhance the precision of prognostic stratification and guide truly individualized therapeutic strategies. In this evolving framework, CPET emerges as an essential gateway to precision medicine in heart failure.

## Data Availability

Data supporting the reported results can be found using the public scientific databases.

## Abbreviations

The following abbreviations are used in this manuscript:

ACE	Angiotensin Converting Enzyme
ARNI	Angiotensin Receptor–Neprilysin Inhibitor
AT	Anaerobic Threshold
AUC	Area Under the Curve
BMI	Body Mass Index
CO	Cardiac Output
COPD	Chronic Obstructive Pulmonary Disease
CPET	Cardiopulmonary Exercise Testing
CRT	Cardiac Resynchronization Therapy
CSA	Central Sleep Apnea
DLCO	Diffusing Capacity of the Lungs for Carbon Monoxide
EOV	Exercise Oscillatory Ventilation
ESC	European Society of Cardiology
eGFR	estimated Glomerular Filtration Rate
HF	Heart Failure
HFimpEF	Heart Failure with improved Ejection Fraction
HFmrEF	Heart Failure with mildly reduced Ejection Fraction
HFpEF	Heart Failure with preserved Ejection Fraction
HFrEF	Heart Failure with reduced Ejection Fraction
HFSS	Heart Failure Survival Score
ICD	Implantable Cardioverter Defibrillator
ISHLT	International Society for Heart and Lung Transplantation
LVEF	Left Ventricular Ejection Fraction
LVAD	Left Ventricular Assist Device
MCS	Mechanical Circulatory Support
MECKI	Metabolic Exercise Cardiac Kidney Index (Score)
MRA	Mineralocorticoid Receptor Antagonist
NIRS	Near-Infrared Spectroscopy
NYHA	New York Heart Association (functional classification)
(VO_2_/HR)	Oxygen Pulse
PaCO_2_	Arterial Partial Pressure of Carbon Dioxide
PetCO_2_	End Tidal Partial Pressure of Carbon Dioxide
PetO_2_	End Tidal Partial Pressure of Oxygen
ppVO_2_	Peak Predicted Oxygen Uptake
RCP	Respiratory Compensation Point
ROC	Receiver Operating Characteristic
RER	Respiratory Exchange Ratio
SGLT2i	Sodium Glucose Co Transporter 2 inhibitor
SHFM	Seattle Heart Failure Model
VD	Dead Space Volume
VE	Minute Ventilation
VE/VCO_2_ slope	Ventilation/Carbon Dioxide Production slope
VO_2_	Oxygen Uptake (Volume of Oxygen consumed)
VO_2_/HR	O_2_ Pulse Oxygen Pulse
VCO_2_	Carbon Dioxide Output
VT	Tidal Volume
WR	Work Rate
